# Reports on the Hospitals of the United Kingdom

**Published:** 1910-05-14

**Authors:** Henry Burdett


### REPORTS
ON THE
### Hospitals of the United Kingdom.

BY SIR HENRY BURDETT, K.C.B., K.C.V.O.

### XLIV.—STAFFORDSHIRE GENERAL INFIRMARY, STAFFORD.

THIS hospital was established in 1766, and the wards were rebuilt and opened in 1897. There are four of them in two pavilions, one on each side of the central portion, which is all that remains of the original hospital. This central portion forms an administration block where all the staff reside, *i.e.* two resident medical officers, the matron, three sisters, and fourteen nurses. The rooms and cubicles are good of their kind, but the arrangement is not ideal, and the committee would be well advised to purchase an adjoining house, now for sale, and convert it into a nurses' home. It should prove a good investment, especially if the committee are wise enough to establish a private nursing staff, and so fulfil to the governors one of the duties which ought to be discharged by every voluntary hospital.

We found everything in order throughout the hospital. The wards are light, airy, and good. The sanitary blocks, though small, are adequate, except in the case of the single wards, where some of the doors are wrongly hung, while the sinks are practically useless. The wards are heated by central stoves, which have not worked well, as they constantly smoke, so that they ought to be properly mended or ended. The out-patient department and the laundry, especially the latter, will have to be reconstructed before long. The hospital contains seventy-five beds, of which only two-thirds are occupied on an average. There are extensive gardens and grounds, in which are placed open-air shelters which the patients use, and we should say that this is a very comfortable resting-place for sick people both in the general and pay wards (three guineas a week).

The advantage of age sometimes is well illustrated by a recent incident in the life of this hospital. The board room contains the library and a few pictures. One picture has recently been proved to be a Gainsborough of value, and will shortly be sold for the benefit of the hospital.

We were taken round the hospital by the matron, Miss Mary Preston, who was trained at the Royal Infirmary, Liverpool, and has her departments well in hand. We also saw one of the resident staff, a New Zealander who is shortly returning home. We have been glad to find in the course of our inspections that other qualified colonials have taken some of the resident appointments in the old country.

### TWO COTTAGE HOSPITALS.

### XLV.—MORETONHAMPSTEAD COTTAGE HOSPITAL.

THIS hospital was opened in 1909, and its existence is mainly due to the generosity of its president, the Hon. W. F. D. Smith. It contains eight beds and two cots, and may fairly be described as a beautiful little hospital. It is perfectly kept and well found throughout. The matron, Miss F. Quilter, is a most active and capable woman, who has everything well organised and in good order. The nursing staff consists of one probationer. There is a certificated midwife who resides in the hospital. Several major operations were performed during the year, and the hospital has the advantage of the services of an ophthalmic surgeon, Dr. McKenzie, of Torquay. More than half the beds were constantly occupied during the year.

The popularity of the hospital and the increasing number of patients make it desirable that the two wards containing three beds each should be extended. This enlargement may easily be effected by taking out the front ends and converting them into wards of six beds each. The cost would be small, and the appearance of the hospital would not be interfered with, as there is plenty of room in front to provide for these extensions.

### XLVI.—ROSS (HEREFORD) COTTAGE HOSPITAL.

THIS is one of the earliest of Cottage Hospitals, and we inspected the original Memorial Cottage Hospital, as it was then called, some thirty-five years ago. The present building was erected about thirty years ago, and is badly planned throughout. In the design and arrangements the architect has failed to display an adequate knowledge of the hygienic and other requirements of a hospital, including air and light. The wards, with four beds and a cot in each, may pass, but the lavatories and sanitary appliances require to be scrapped and new ones erected. There is a dispensary attached to the hospital, and the provision made for seeing the patients, and the dispensary itself, which has no outside window and is practically without ventilation, is the worst we have come across anywhere. It is essential that these grave defects and the supply of adequate bath accommodation should be taken in hand by the authorities. The dispensary should be rearranged and reconstructed without a moment's avoidable delay.

The wards are well kept, and the patients are contented and happy. The grounds are attractive, and the matron, who is a skilled gardener, has made the gardens very charming.

The operation theatre and its fittings, given by an American lady, are things to see and wonder at. Nothing more remarkable is to be found in a hospital of this type.

The **Manchester Children's Hospital, Pendlebury,** will be reported on next week.

